# Acute Lumbar Burst Fracture Treated by Minimally Invasive Lateral Corpectomy

**DOI:** 10.1155/2013/953897

**Published:** 2013-03-24

**Authors:** Rodrigo Amaral, Luis Marchi, Leonardo Oliveira, Thiago Coutinho, Luiz Pimenta

**Affiliations:** ^1^Department of Minimally Invasive Surgery, Instituto de Patologia da Coluna, Vergueiro Street, 1421, Sala 305, Torre Sul, Paraíso, 04101-000 São Paulo, SP, Brazil; ^2^Department of Imaging Diagnosis, Universidade Federal de São Paulo, Napoleão de Barros Street, 800, Vila Clementino, 04024-002 São Paulo, SP, Brazil; ^3^Department of Neurosurgery, University of California, San Diego, 200 West Arbor Drive, CA 92103, USA

## Abstract

Burst fractures in acute spinal traumas are a difficult problem to solve. Different approaches and techniques have been utilized, but with high incidence of morbidity and mortality, besides unsatisfactory clinical and radiological results. Mini-open approaches recently emerged and have been shown to be safe and effective in the treatment of several spinal conditions. Here we report a case of acute lumbar burst fracture at L2 treated by minimally invasive true lateral approach posteriorly instrumented with percutaneous pedicle screws. The minimum disruptive access in addition to a rigid construction allowed a lumbar corpectomy without the morbidity of standard open approaches, lowering surgery costs and accelerating the patient recovery with successfully clinical and radiological results.

## 1. Introduction

The treatment of burst fractures in acute spinal traumas represents a complex decision-making process [1]. Some considerations must be analyzed in order to decide which way to proceed. Posterior-only approaches are widely utilized, but failure to maintain the sagittal plane correction has been observed [2]. However, when the main goal is decompression of the spinal canal and stabilization of the segment, the anterior approach should be the technique of choice [3].

This technique provides direct decompression of the neural structures, providing an appropriate anterior support and load sharing with the use of a vertebral body replacement device [4]. However, excessive blood loss, damage of the abdominal wall, permanent injuries in diaphragm, and incisional pain are related to the anterior approach [5]. 

Recently, mini-open approaches to the thoracolumbar spine have been shown to be safe and effective in the treatment of several spinal conditions including vertebral fractures, with minimum blood loss, muscle splitting, and pain [6]. Here we report a mini-open true lateral transpsoas approach for lumbar corpectomy supplemented with percutaneous pedicle screws in the treatment of an acute lumbar burst fracture. 

## 2. Case Report

HK, 55-year-old male, was involved in a fall from 3 meters height. The patient complained of immediate back pain with some irradiation numbness and weakness to the right leg. He was initially admitted in a countryside hospital and further transferred to São Paulo, SP, Brazil, to be followed up by our group. 

Initial physical examinations showed tenderness on palpation of the back at the level of L2 spinal process. The patient had anterior tight numbness and weakness in knee extension, with motor strength grade 4, an ASIA motor score of 98 (normal = 100), sensitive score of 110 (normal = 112), and D in Frankel scale. Moreover, the patient was conscious and has no additional injuries.

Initial anteroposterior and lateral X-rays evidenced a burst fracture of L2 vertebra with an increased distance between the pedicles, a loss of 32% of the vertebral heights, and increased local kyphosis in 11.7°, besides a 4.4 mm retrolisthesis. Cross-sectional CT scan showed an intracanal fragment that filled around 80% of the canal area, in addition to a transverse process fracture on both sides.

**Figure 1:**
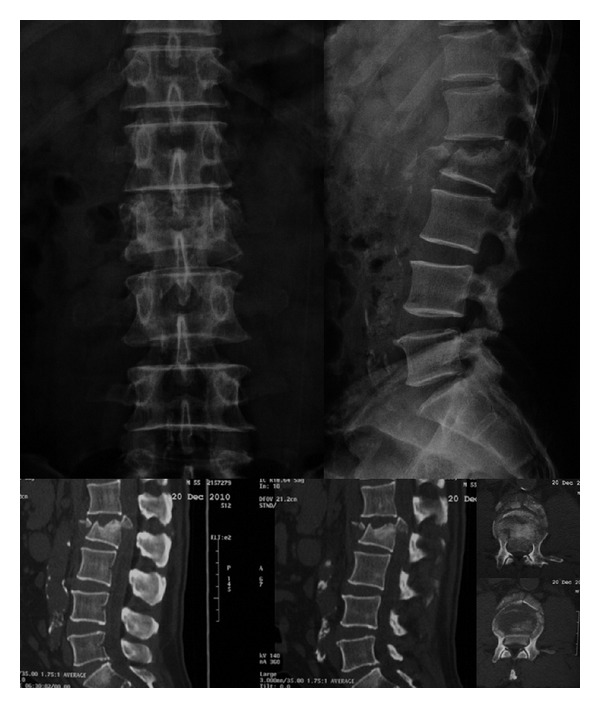
X-ray and CT scan showing a burst fracture on L2 vertebra with a fragment inside the spinal canal.

The authors classified this fracture as a Type C burst fracture according to Denis classification [7] and A3 according to AO/Magerl classification (Burst fracture) [8] (Figure 1).

The patient was brought to OR on day 1 after injury and the lateral transpsoas approach (XLIF) was chosen in order to perform the corpectomy and fusion due to its minimal disruption of the soft tissue, direct visualization of the vertebral body, neural elements and spinal canal, and the possibility to insert an expandable vertebral body replacement device in a minimal invasive fashion.

The patient was placed and taped in a true lateral position in a radiolucent table. A mark was made on the skin based on fluoroscopy that identified the fracture level. Then, a 7 cm incision was made and the retroperitoneal space was dissected, as previously described [9]. 

To cross the psoas muscle in a safe position, neuromonitoring was obtained using a free-run electromyography (EMG) with somatosensory and motor evoked potentials (SSEP & MEP, NeuroVision M5, NuVasive, Inc., San Diego, CA, USA). Real-time neuromonitoring was used during passage through the psoas muscle, retractor expansion, and instrument implantation. Sequential tube dilation was used to distract the psoas muscle until docking the expandable retractor (MaXcess, NuVasive, Inc.) over the disc space. Initially, inferior and superior discectomies in the upper and lower levels of the fracture and coagulation of segmental vessels were performed. The corpectomy was then conducted working within the space defined by the retractor on lateral projection from the upper to the lower disc space, the retropulsed fragments were mobilized, and the spinal canal was decompressed using standard instruments and techniques.

**Figure 2:**
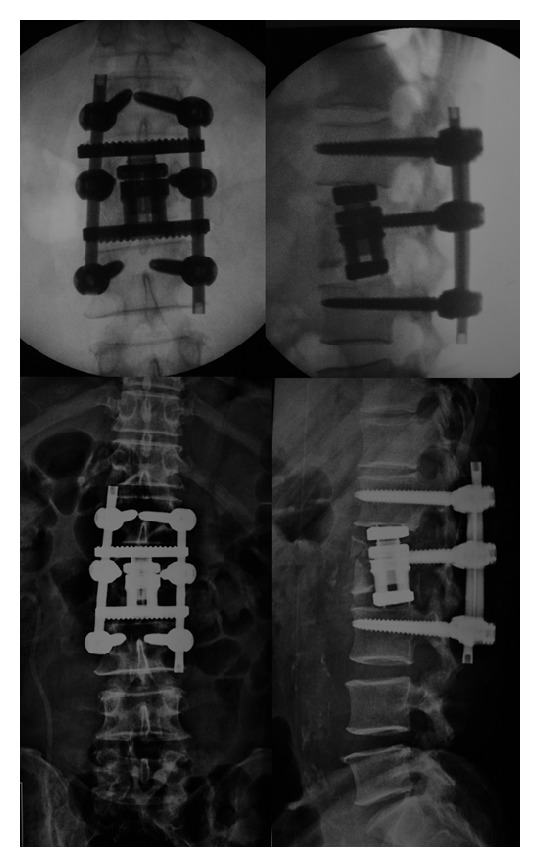
Intraoperative fluoroscopy and immediate postoperative X-ray showing implant position and fracture reduction.

 Vertebral body replacement was performed using a wide-footprint expandable Ti cage (XCore, NuVasive, Inc.). Autograft was used inside and outside the cage from the vertebral body itself. The cage was expanded reducing partially the local kyphosis using the vertebral endplates of L1 and L3 as points of fixation. Due to the wide footprint, the cage rests on the ring apophysis, enhancing biomechanical support. After closing the operative wound in a standard fashion, the patient was positioned in ventral decubitus and a supplemental internal fixation was done utilizing posterior percutaneous pedicle screw fixation (MIP, MDT, Inc.) one level above and one below fracture (Figure 2).

The overall duration of the procedure was 300 minutes, with intraoperative blood loss of 350 mL. Patient did not need to stay in the intensive care unit, no blood transfusion was required, and the total length of hospital stay was only one day. Standing position and ambulation were also performed on the first postoperative day, before hospital discharge.

**Figure 3:**
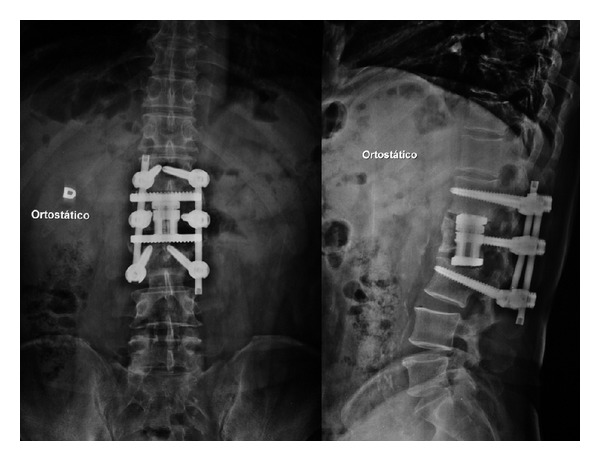
AP and lateral X-rays showing good coronal and sagittal alignments 12 months after surgery.

**Figure 4:**
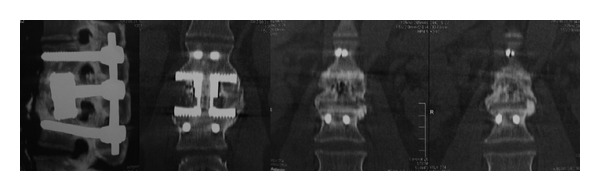
CT scan showing solid fusion 24 months after the procedure. The device design promotes the ossification inside and outside of the prosthesis.

The patient was evaluated throughout 24 months, showing improvement in clinical and radiological conditions. The 12-month X-rays show improvement in sagittal and coronal alignments (Figure 3), while solid fusion was achieved 24 months after surgery (Figure 4), maintaining favorable clinical and radiological statuses.

## 3. Discussion

Thoracolumbar fractures are very common in spine practice, and there are several therapeutic options for its treatment [10–13]. When surgery is needed, its goals are to decompress the spinal canal and neural elements to facilitate neurologic recovery, restore and maintain the vertebral body height for coronal and sagittal alignments, generate a rigid construction to allow early ambulation and rehab, and prevent deformity progression and unbalance of the spine to avoid neurological deficit, while limiting the number of instrumented segments fused [14–17]. 

The anterior approach to the burst fracture is indicated for cases with severe canal compromise and kyphotic deformity [18, 19]. However, the operative risk is relatively high and includes excessive blood loss, permanent diaphragm failure, abdominal wall injuries, pulmonary complications, and prolonged incisional pain with high infection rates [20, 21]. Mini-open anterior approaches proved to be a less invasive but still open alternative to access the thoracolumbar spine, with the 3-dimensional view of the structures that facilitates the surgical procedure and corpectomy cage insertion [6]. 

Regarding perioperative factors, Lu et al. [20] found a mean operative time of 445 minutes in patients with anterior-only corpectomy, with a mean EBL of 1506 mL. When adding a posterior approach, mean operative time was 729 minutes with a mean blood loss of 3154 mL. Using a lateral approach to the spine with percutaneous pedicle screw supplementation (one level above and one below), we were able to perform a 1-level corpectomy in 300 minutes with only 350 mL of blood loss, with minimal soft tissue dissection and muscle splitting, which enabled a decreased length of hospital stay and faster return of normal daily living.

One of the major difficulties in anterior approaches is to reduce the kyphotic fractures, being long fixation using pedicle screws (2 above and 2 below fracture) the most adequate to this indication [19, 22]. Using an expandable vertebral body replacement device, we were able to restore the sagittal alignment of the spine without the need of extent posterior manipulation, saving motion segments. Differently from nonexpandable corpectomy devices [23], particularly with cylindrical cages that rest inside the border of the apophyseal ring [24], the lateral approach also permits the insertion of a wider footprint device that reaches apophyseal ring bilaterally, enhancing biomechanical stability and preventing subsidence, kyphosis progression, and restenosis [25–27]. 

## 4. Conclusion

The miniopen lateral approach offers the advantages of minimally invasive surgery (MIS) for lumbar corpectomy without the morbidity of standard open approaches, facilitating a wider cage insertion, reducing operative time, blood loss, and adjacent tissue and muscle injuries, lowering surgery costs, and accelerating the patient recovery with the same or better clinical and radiological results of traditional techniques.
